# Evaluating Public Participation in a Deliberative Dialogue: A Single Case Study

**DOI:** 10.34172/ijhpm.2022.6588

**Published:** 2022-02-28

**Authors:** Tiffany Scurr, Rebecca Ganann, Shannon L. Sibbald, Ruta Valaitis, Anita Kothari

**Affiliations:** ^1^School of Health Studies, Faculty of Health Sciences, Western University, London, ON, Canada.; ^2^School of Nursing, Faculty of Health Sciences, McMaster University, Hamilton, ON, Canada.; ^3^Schulich Interfaculty Program in Public Health, Schulich School of Medicine and Dentistry, Western University, London, ON, Canada.; ^4^Department of Family Medicine, Schulich School of Medicine and Dentistry, Western University, London, ON, Canada.

**Keywords:** Community Engagement, Stakeholder Consultation, Knowledge Translation, Public Engagement, Public Involvement, Deliberative Dialogue

## Abstract

**Background:** Deliberative dialogues (DDs) are used in policy-making and healthcare research to enhance knowledge exchange and research implementation strategies. They allow organized dissemination and integration of relevant research, contextual considerations, and input from diverse stakeholder perspectives. Despite recent interest in involving patient and public perspectives in the design and development of healthcare services, DDs typically involve only professional stakeholders. A DD took place in May 2019 that aimed to improve the social environment (eg, safety, social inclusion) and decrease social isolation in a rent-geared-to-income housing complex in a large urban community. Tenants of the housing complex, public health, primary care, and social service providers participated. This study aimed to determine how including community tenants impacted the planning and execution of a DD, including adjustments made to the traditional DD model to improve accessibility.

**Methods:** A Core Working Group (CWG) and Steering Committee coordinated with researchers to plan the DD, purposefully recruit participants, and determine appropriate accommodations for tenants. A single mixed-methods case study was used to evaluate the DD process. Meeting minutes, field notes, and researchers’ observations were collected throughout all stages. Stakeholders’ contributions to and perception of the DD were assessed using participant observation, survey responses, and focus groups (FGs).

**Results:** 34 participants attended the DD and 28 (82%) completed the survey. All stakeholder groups rated the overall DD experience positively and valued tenants’ involvement. The tenants heavily influenced the planning and DD process, including decisions about key DD features. Suggestions to improve the experience for tenants were identified.

**Conclusion:** These findings demonstrate the viability of and provide recommendations for DDs involving public participants. Like previous DDs, participants found the use of engaged facilitators, issue briefs, and off-the-record deliberations useful. Similarly, professional stakeholders did not highly value consensus as an output, although it was highly valued among tenants, as was actionability.

## Background

**Key Messages**

** Implications for policy makers**

- Involving members of the public in deliberative dialogues (DDs) brings new perspectives to policy discussions and was highly valued by all stakeholder groups. Inclusion of those affected by the issue increased community trust in the initiatives undertaken in their name.
- Public participants require unique accommodations in DDs and should be distinctly considered during the phases of planning and evaluation.
- Providing public participants with an orientation session prior to a DD increased their feelings of preparation, comfort, and value when at the table with professional stakeholders.
- Public participants contributed tacit knowledge and experience differently than other stakeholders, through impactful personal narratives and stories, requiring skilled facilitation to recognize and navigate.

** Implications for the public**

Deliberative dialogues (DDs) provide a promising forum for public voices to be heard and integrated into healthcare service delivery and design. The conduct and principles of DDs have previously been studied as a means of enabling equity-focused and inclusive discussions. We deepen this exploration into how these features support the inclusion of public voices and how they can be modified to increase accessibility. This study demonstrates to researchers and policy-makers that the involvement of public participants in DDs is feasible and provides a guideline for accommodations and adjustments that should be anticipated. With this information, those organizing DDs can be better prepared to engage members of the public, leading to more efficient processes and positive experiences of inclusion for public participants, if their accommodations are appropriately considered and anticipated.

###  Deliberative Dialogues

Deliberative dialogues (DDs) are a type of policy dialogue that have been widely explored as a viable knowledge translation and research uptake strategy, as they allow organized dissemination and integration of relevant research, contextual considerations, and input from diverse stakeholder perspectives on an issue.^1^ DDs are advantageous because they bring together stakeholders from multiple sides of an issue to discuss the applicability and transferability of research, resulting in transformative discussion and the potential to influence policy development.^1-3^

DDs are distinguished from other stakeholder dialogues, such as round tables, by three features: (*i*) participants represent more than one stakeholder group (eg, policy-makers, researchers, service providers); (*ii*) research evidence provides input into the dialogue; and (*iii*) tacit knowledge and experience from participants provide input into the dialogue.^2,3^ Typically, a DD is planned by researchers in collaboration with a steering or advisory committee comprised of affected stakeholders.^2,4^ Most DDs are conducted over three separate phases: pre-DD, DD, and post-DD. Planning begins two to six months before the deliberation, and post-DD activities (including participant debriefing) and evaluation typically occur for up to six months post-DD.^5,6^ Research input is usually synthesized in the form of an issue brief (sometimes called an evidence brief) compiled by the research team and disseminated to participants a week or two before the DD.^7^ These briefs summarize current literature and present relevant solutions and research findings which are ideally integrated into the DD discussions.^2^

###  Public Participants as Stakeholders

A key purpose of DDs is to gain variable input, opinions, and experiences of those impacted by problems and involved in implementing solutions.^8^ A group of scholars has demonstrated that DDs can be used to foster equity-focused engagement from these stakeholders.^9^ The key features and conduct of DDs, namely their diversity, preparation of participants, and collective commitment, are considered to advance inclusive and equitable research through principles of relational engagement and accountability.^9^ Given the suitability of DDs to support equitable engagement, and as engagement of the public becomes increasingly popular in research and health services decision-making, exploring the integration of public perspectives in DDs becomes even more relevant.^10,11^

Although Boyko^2^ suggests that members of the public are viable stakeholders, most DDs include only professional stakeholders such as those involved in research, policy, or decision-making.^3,5,7^ To date, we are aware of only a small number of published academic studies that have involved patients or public participants in a DD among other stakeholders, one including young adults with cerebral palsy and their family members, and the other engaging women living with HIV.^6,12^ O’Brien and colleagues^12^ briefly reported three strategies that were incorporated to balance power dynamics between stakeholders: (*i*) setting rules of engagement, (*ii*) dividing patients and providers into separate small groups, and (*iii*) recruiting public participants with previous participatory research experience. However, while these studies provide a budding understanding of DDs that include the public, they focused and reported more heavily on the DD topic and outcomes than the process of involving the public, and thus did not explore the impact that public members’ involvement had on the planning or outcomes in depth. To advance the field, further research is necessary to evaluate DD methodology with a focus on the adjustments, accommodations, and impact on stakeholder relationships resulting from inclusion of the public.

There is consequently a methodological gap in the literature for researchers who want to include public participants. It is recognized in the DD literature that key design features are not one-size-fits-all, making it difficult to compare contextual features among DDs for the purpose of planning future DDs.^3^ Without existing examples of public inclusion or comprehensive reviews demonstrating which and how key DD features can be adjusted and extrapolated from previous contexts, research exploring the inclusion of public participants in DDs is necessary to expand the evidence base.

###  Research Objective

 This study aimed to evaluate the three phases of a DD that involved public participants – in this case, community tenants – as a stakeholder group. Some were involved in the planning process, dialogue, and post-dialogue evaluation, whereas others participated in the dialogue alone. We addressed the following questions:

1. How is the traditional DD model adjusted to include diverse views from decision-makers and community tenants?
2. How do all stakeholders, including community tenants, perceive and respond to the inclusion of community tenants in the DD?
3. What impact does the inclusion of community tenants in the collaboration have on the process of planning the DD and course of the DD discussions?

## Methods

A mixed-methods single case study approach was used. Case study methodology allowed us to examine howthe inclusion of community tenants impacted the DD process without necessitating the separation of the phenomenon from the context.^13-15^

###  Setting

This project was a sub-study of the two-phase INSPIRE-PHC project exploring the implementation and evaluation of an intervention to foster collaboration among primary care and public health (hereafter referred to as the INSPIRE project). This project took place in a large urban community in Southwestern Ontario, Canada to improve neighbourhood health in a rent-geared-to-income housing complex with 565 tenants. In phase one of the INSPIRE project, a Core Working Group (CWG) was established to identify gaps in neighbourhood data and co-create a Community Health Profile about the population living in the complex and surrounding neighbourhood.^16^ In phase two of the INSPIRE project, a DD was planned and implemented in collaboration with the CWG to address social isolation and improve the social environment in the housing complex. The CWG consisted of the housing complex tenants, service providers from the city housing corporation (hereafter referred to as city housing), public health and primary care, and researchers. All group members (save the researchers) served or lived in the housing complex. This current study evaluates the planning and implementation of the DD.

Based on a document review of project files and the Community Health Profile, a narrative summary of the context of this case is represented in Supplementary file 1. This summary describes the demographics, history, and culture of the housing complex.

###  The Planning Process

 Three teams collaborated to plan the DD: the research team, an existing Steering Committee, and the CWG; members of the research team attended all meetings and provided administrative support. The Steering Committee was a city housing-led committee that consisted of tenants, staff members and/or administrators from city housing, public health, the Ontario Disability Support Program, primary care providers, and researchers. Over the course of five months (January 2019 to May 2019) regular face-to-face meetings were held with each of the three teams to plan the DD.

 With feedback from the CWG, the research team developed a 32-page issue brief (including a five-page executive summary) of synthesized research evidence that was distributed to participants before the DD. It included results of four rapid evidence syntheses conducted by the research team to address four main issues outlined in the Community Health Profile and identified by the CWG (mental health and addictions; engaging tenants in decision-making; improving communication between tenants and service providers; and fostering social interaction between tenants), and presented potential research-based solutions. To ensure it was accessible to the diverse group of stakeholders, researchers aimed to write the issue brief at a fourth grade reading level and avoid the use of jargon. It was sent to the CWG for feedback four weeks prior to the DD and revisions were suggested at the subsequent CWG meeting.

###  Participants

DD participants were composed of two groups: professional stakeholders and tenants. ‘Professional stakeholders’ refers to those impacted by or connected to the issues under the purview of their organization or profession. They were purposefully selected by a sub-committee of CWG members, including service providers, tenants, and the research coordinator, familiar with relevant professional roles in the community. The sub-committee considered roles they believed would be valuable to have represented and members suggested individuals or organizations. A list of potential participants was sent to the research team to determine whether individuals were appropriate participants and ensure all relevant roles had been considered. These roles included service providers, policy-makers, city counsellors, city housing staff, and health and social care providers with experience serving the housing complex. When possible, senior-level managers from organizations were chosen based on their position to make decisions and influence change, as is a key consideration of recruitment in DDs.^5^ Professional stakeholders were invited via email two and a half months before the DD. If any declined, someone with a similar professional role was invited. Tenant participants were recruited face-to-face by a service provider and tenant who were CWG members and familiar with tenants in the complex. Selection criteria, determined by the CWG sub-committee, included tenants who were familiar with the available resources, supports, and challenges faced in the housing complex; have good communication skills; could speak to the needs of other tenants beyond their own personal experiences; and represented diverse ages, genders, races, employment status, and disabilities. Tenants were offered CAD$15 per hour in the form of gift cards for their participation in the pre-DD orientation session and DD (totalling $100), and $20 for participation in either focus group (FG).

###  The Deliberative Dialogue

 One week before the DD, participants were emailed a copy of the agenda, the Letter of Information and Consent, the Community Health Profile, a list of anticipated participants, and the issue brief. To ensure that tenants felt prepared and comfortable attending the DD, the researchers held a two-hour orientation for tenants. This session occurred two weeks prior to the DD and attendance was mandatory for tenants who wished to participate in the DD to ensure they had foundational knowledge of the research and an opportunity to ask questions. At the session, the research team obtained informed consent to participate in the DD, provided a copy of the issue brief, explained the purpose of the DD, briefly explained each of the four issues, and described the evidence-based solutions presented in the issue brief.

The DD was held in May 2019 and lasted 4.5 hours over one afternoon; 43 people attended, including nine researchers who facilitated or recorded notes. Participants were assigned to one of four small groups with seven to nine discussants. Small groups were assigned by a researcher with knowledge of the relationships between service providers and tenants, such that tensions from pre-existing relationships were minimized and stakeholder groups were evenly represented at all tables. With help from a facilitator, each small group discussed one of the four issues and attempted to reach consensus on the most appropriate solution. The small groups took turns presenting this solution to the larger group in a plenary session facilitated by a researcher. Returning to the same small groups, a second of the four topics was discussed, and consensus was again presented to the larger group. Following the final large group discussion, each participant voted for their favourite solutions in a dotmocracy. In this activity participants received three colour-coded dots to reflect their stakeholder group and placed them on their favourite solution(s) from the plenary sessions, which were listed on a large piece of paper posted to the wall, and the time frame they believed was reasonable to implement that solution (1-2 months, 6 months, 1 year, or 2-3 years). Before leaving, all participants were asked to complete a survey. The full agenda is available in Supplementary file 2.

###  Data Collection

 Data were collected over a twelve-month period (December 2018 to November 2019). There were three main sources of data: meeting minutes, post-DD participant surveys, and FGs.

 Collectively, these data reflected a range of perspectives from the members of the Steering Committee, CWG, research team, and DD participants. Field notes maintained throughout the research process were integrated into the qualitative analysis.

####  Meetings

 Meeting minutes and field notes were collected during all team meetings over the course of the DD process. This included two Steering Committee meetings, five CWG meetings, and 14 research team meetings.

####  Post-dialogue Participant Surveys

At the end of the DD participants were asked to complete a 29-item paper survey about their experiences with and opinions of the key features of the DD. Participants first indicated the stakeholder group to which they belonged so answers could be categorized and compared. The survey consisted of: (*i*) open-ended questions (eg, “How did you feel about [stakeholder group]’s participation in the DD?”); (*ii*) a question asking participants to rate the overall dialogue from a score between 1 (low) and 10 (high); and (*iii*) questions asking participants how much they disagree or agree with statements about key features of the DD with the option to explain their response (eg, “It was helpful that consensus was encouraged in the DD”) with the options of strongly disagree, disagree, neutral, agree, and strongly agree. The survey incorporated key components of Boyko and colleagues’^5^ questionnaire. See Supplementary file 3 for examples of key features addressed in the survey.

####  Focus Groups

 FGs were conducted by research team members, lasted approximately one hour, and included key DD participants as identified by the facilitators of the small group sessions, based on engagement in and contribution to the DD discussions. Three FGs were held one month after the DD; two with professional stakeholders (n = 2; n = 3) and one with tenants (n = 10). None of the participants involved in the one-month FGs were members of the CWG so that perceptions and expectations expressed about the DD were not influenced by knowledge about the desired outcomes. Two FGs were held six months after the DD, one with professional stakeholders (n = 6) and one with tenants (n = 5). FGs were facilitated by a researcher, audio-recorded, and professionally transcribed verbatim.

###  Data Analysis 

All qualitative data, including meeting minutes, field notes, open-ended survey responses, and FG transcripts were uploaded to NVivo 12 and coded using inductive thematic analysis at a semantic level.^17^ Quantitative data from the post-DD participant surveys were analyzed using descriptive statistics.^18^

 The mean and mode survey response of each stakeholder group was calculated for questions that prompted participants to indicate agreement on a scale of strongly disagree (coded as 1) to strongly agree (coded as 5).

## Results

Themes that were identified during data analysis were grouped into four main categories. First, qualitative and quantitative *survey results* are reported together. Three themes were identified within the qualitative data: *tenant impact on planning and key decisions, tenant impact on deliberative discussions, *and *tenant impact on the DD process. *Each of these themes are further broken down into sub-themes.

###  Survey Results

 In total, 28 of 34 DD participants completed the survey (response rate = 82%). These participants identified as tenants (n = 13; 46%), social services staff/manager/director (n = 5; 18%), public health staff/manager/director (n = 3; 11%), and other (n = 7; 25%). “Other” included hospital and home care workers, a funder, and primary care staff.

The DD received an overall mean rating of 8.46 out of 10 across all respondents. The most common response across all stakeholder groups was 4 out of 5 (agree) for all questions pertaining to the favourability of DD features. The results of the survey are presented below to reflect participants’ feedback about the*favourability of key features*, which are ranked within and across stakeholder groups, the *accessibility *of and* preparation* for the DD, compared across stakeholder groups, and the *perceived value of participation*, which summarizes how participants perceived their own contributions to the DD and how valued each stakeholder group felt by other participants.

####  Favourability of Key Features

The mean response of all participants rated the most favourable feature as *the use of an engaged facilitator* to assist with the DD and the least favourable was that *the right people were involved to think about the health and wellness for the tenants. The use of an engaged facilitator* was the most favourable feature for all stakeholder groups but social services, who rated* the use of an issue brief *more favourably. Tenants ranked *encouraging consensus* the highest among all stakeholder groups (4.46).

On average, public health gave the highest mean scores across all features (4.5), although the “other” group rated the DD highest overall (with a mean score of 8.86/10). Social services gave the lowest mean score across all features (4.19), rated four of the six key features less favourably than any other stakeholder group, and rated the DD lowest overall (7.6/10). Tenants rated the DD 8.69/10 overall and gave a mean score of 4.41 across all features. See Table 1 for a summary of results.

**Table 1:** Survey Results Addressing Key Features

**Survey Question Addressing Key Feature**	**Overall Mean** **(n = 28)**	**Overall** **Rank**	**Tenant ** **Mean** **(n=13)**	**Rank Among Tenants**	**SS Mean (n=5)**	**Rank Among SS**	**Public Health Mean (n=3)**	**Rank Among Public Health**	**Other Mean (n=7)**	**Rank Among Other**
It was helpful to have the DD informed by the pre-circulated research summary [issue brief].	4.48	2	4.64	1	4.50	1	4.33	2	4.29	3
In the DD, the right people were involved to think about health and wellness for the tenants.	4.11	5	4.15	5	4.00	3	4.33	2	4.00	4
It was helpful to have the opportunity to discuss different features of the problem, including (where possible) how it affects particular groups.	4.18	4	4.15	5	3.80	4	4.33	2	4.43	2
It was helpful to have an engaged facilitator to assist with the DD.	4.61	1	4.62	2	4.40	2	5.00	1	4.57	1
It was helpful that the DD allowed for frank, off-the-record deliberations.	4.48	2	4.42	4	4.40	2	5.00	1	4.43	2
It was helpful that consensus was encouraged in [the] DD.	4.19	3	4.46	3	4.00	3	4.00	3	3.83	5

Abbreviations: SS, social services; DD, deliberative dialogue. Mean scores on a scale of 1 (strongly disagree) to 5 (strongly agree) were calculated for each survey question within each stakeholder group and overall across all participants. The statements were ranked within each stakeholder group from most to least agreeable according to the calculated means.

####  Accessibility and Preparation

Table 2 displays the mean stakeholder responses to questions about accessibility and preparation. The accessibility of the venue was rated 4.58 overall. Tenants found the venue least accessible of all stakeholder groups, with one tenant giving the lowest score of 2/5 (disagree). One respondent recommended taxi vouchers be offered to tenants with mobility barriers.

**Table 2:** Survey Results Addressing Preparation and Accessibility

**Survey Question**	**Overall Mean** **(n = 28)**	**Tenant ** **Mean** **(n=13)**	**Rank Among Tenants**	**SS Mean (n=5)**	**Rank Among SS**	**Public Health Mean (n=3)**	**Rank Among Public Health**	**Other Mean (n=7)**	**Rank Among Other**
It was easy for me to get to (attend) the DD.	4.58	4.50	4	4.75	1	4.67	2	4.57	3
I feel that I was well prepared to participate in the DD.	4.35	4.42	2	4.75	1	4.00	4	4.14	3
The discussion at the DD was easy for me to understand.	4.44	4.67	1	4.40	2	4.00	4	4.29	4
I could easily read and understand all of the documents needed for me to participate in the DD.	4.39	4.23	4	4.80	1	4.33	3	4.43	2

Abbreviations: SS, social services; DD, deliberative dialogue. Mean scores on a scale of 1 (strongly disagree) to 5 (strongly agree) were calculated for each survey question within each stakeholder group and overall. The statements were ranked across stakeholder groups from most to least agreeable according to the calculated means.

 Social services felt most prepared to participate in the DD (4.78) and those with positive feedback indicated that they felt prepared by the materials sent in advance. Public health collectively felt least prepared to participate (4.00) with one respondent indicating that they felt they had little to contribute to the discussion. Tenants indicated that they felt prepared by meeting with a researcher to explain materials and consent before the DD, reading the Community Health Profile, and due to their “advantage of experience.”

 Tenants rated the discussion the easiest to understand of all stakeholder groups (4.67) and found the open discussion and small groups helpful. However, when asked about the ease with which respondents could read and understand the documents provided to participate in the DD, tenants had the lowest mean (4.23) and social services the highest (4.80). One tenant reported that they had trouble understanding the meaning of some words and one “other” respondent suggested that the issue brief be written at a lower reading level and with larger font.

####  Perceived Value of Participation

Overall, tenants felt the most heard and valued of all stakeholders (4.62) with the most common response of 5 (strongly agree) to both relevant statements. All but one tenant agreed or strongly agreed that they felt valued and heard when expressing ideas. Tenants reported feeling that others were listening; one indicated feeling that their participation was necessary because their experiences were unlike others who were present. However, other stakeholders indicated that they did not have as much to contribute or purposefully contributed less to allow tenants more time. Public health agreed least overall with feeling their ideas were heard (4) and felt the least valued (3.67). Results are displayed in Table 3.

**Table 3:** Survey Results Addressing Respondents’ Perceived Value of Their Participation

**Survey Question**	**Overall Mean** **(n = 28)**	**Tenant ** **Mean** **(n=13)**	**Rank Among Tenants**	**SS Mean (n=5)**	**Rank Among SS**	**Public Health Mean (n=3)**	**Rank Among Public Health**	**Other Mean (n=7)**	**Rank Among Other**
I felt that my ideas were heard at the DD.	4.36	4.62	1	4.20	2	4.00	4	4.14	3
I felt that my participation in the DD was valued.	4.21	4.46	1	4.00	3	3.67	4	4.14	2

Abbreviations: SS, social services; DD, deliberative dialogue. Mean scores on a scale of 1 (strongly disagree) to 5 (strongly agree) were calculated for each survey question within each stakeholder group and overall. The statements were ranked across stakeholder groups to demonstrate which stakeholders found the statement most to least agreeable according to the calculated means.

###  Tenant Impact on Planning and Key Decisions

The DD was planned with input from the Steering Committee, CWG, and research team over the course of five months. Table 4 is based on research findings from meeting minutes and field notes and depicts which teams had input in the decision-making process about key features of the DD. Table 4 depicts which topics were significantly discussed or influenced by each team over the course of their meetings. Tenants influenced key decisions either through direct input (via tenants in the CWG) or because their anticipated participation shaped decisions made by the teams throughout the DD process.

**Table 4:** Key Team Inputs Into Deliberative Dialogue Planning

**Decision Impacted**	**Research Team**	**CWG**	**Steering Committee**
Topic of DD and issues of focus	✓	✓	✓
Goal of DD	✓	✓	✓
Agenda	✓	✓	
Evidence syntheses	✓		
Issue brief	✓	✓	
Participant selection for DD		✓	✓
Communication with participants		✓	
Pre-DD orientation (purpose, process)	✓	✓	
DD facilitation process	✓	✓	
Small group questions to prompt discussion	✓	✓	
Dotmocracy for prioritization of actions	✓	✓	
Post-DD action	✓	✓	✓
Responsibility for solutions			✓

Abbreviations: CWG, Core Working Group; DD, deliberative dialogue. This table depicts research findings about the key features and details each team discussed and contributed to in a decision-making capacity. Note that all researchers were members of the CWG and a minimum of two researchers attended the Steering Committee meetings. *Communication with participants* refers to decisions made about what documents to send to participants of the DD and when. *Responsibility for solutions* refers to the assignment of leaders to take responsibility for the top six solutions identified at the DD.

Table 5 displays the adjustments made to the traditional DD model to enable inclusion of community tenants. These adjustments were informed by CWG tenants and service providers, as well as researcher observations made throughout the INSPIRE study.

**Table 5:** Changes to the Traditional Deliberative Dialogue Process and Features

**Phase **	**Impact **	**Reason for Adjustment**	**Consequences?**
Pre-DD (Planning)	a. Added pre-DD orientation session (2 hours)	To increase tenant confidence with issue brief and DD process	Extra time commitment for researchers, tenants Did not complete agenda; required one-on-one meetings with tenants to sign the letter of information and consent and discuss the issue brief
	b. Issue brief language, complexity, presentation	To increase tenant confidence with/understanding of material and process Suggested by CWG during issue brief edits	Length of issue brief increased
	c. Tenant participant recruitment closer to DD, extra recruitment	To decrease time between commitment to participate and DD Advised by CWG that some tenants may not participate without notifying research team in advance	All participants attended; 3-4 more than anticipated Required extra compensation for participants and alternative planning for table seating
	d. Careful planning of small group seating	To separate tenants and service providers with challenging or personal pre-existing relationships, where possible To avoid hostilities between tenants with history of conflicts	Required knowledge of tenant and stakeholder relationships
DD	a. Agenda shortened (4.5 hours); DD held in afternoon; more breaks included	Accessibility to tenants with chronic conditions Tenants may be less accustomed to sitting through meetings for long periods of time	Not all topic issues could be covered by all groups
	b. Relaxation room	Private space for participants to use if at any point they needed a break from discussing potentially sensitive topics	n/a
	c. Separate area to complete surveys (*suggested*)^a^	Some tenants needed help writing survey responses; answers were communicated out loud within ear shot of stakeholders and facilitators	Members of the research team must be available during survey completion Using supports and the lack of privacy may have led to more moderate/less truthful answers
Post-DD (Evaluation)	a. Separate FGs for professional stakeholders and tenants	Power dynamic	Tenants spoke more freely Extra time commitment for researchers to host 2x the FGs

Abbreviations: CWG, Core Working Group; DD, deliberative dialogue; n/a, not available; FGs, focus groups. This table describes changes made to the typical DD process and features to accommodate the inclusion of community tenants. Accommodations were made in response to suggestions by the CWG or observations made by the research team. ^a^ Suggested accommodation based on researcher observations/field notes; not implemented in this DD.

####  Actionability

 Actionability was one of the most important influences on how decisions were made and was the most prevalent concern among tenants. Tenants in the CWG reported that their housing complex had been involved in three research projects over nine years, none of which they felt resulted in action or improvements. Consequently, during meetings some tenants openly expressed reservations about trusting the commitment of the research team before seeing action or change. This sentiment was echoed in the tenant FG: “you have to decide whether you engage and spend a lot of time for it to come to nothing” (Tenant FG 1). Both tenants and professional stakeholders expressed that quick, actionable solutions that demonstrated change and improvement were necessary to maintain the participation, support, and trust of the tenant community:

“*My biggest reason for coming [to the deliberative dialogue and focus group] is action. And I think that action is what’s needed…and listening to the tenant rather than just lip service, telling us what ‘we’re going to do’”* (Tenant FG1).

 Ultimately this focus on actionability led to the decision that consensus would be a goal of the DD and a dotmocracy would be used to vote on solutions. The top solutions could then be brought to the Steering Committee to identify individuals responsible for each. The drive for consensus also shaped the questions asked to the small groups to encourage thoughts about actionable solutions with less abstract discussion. For example, participants were prompted to refer to specific reasons why solutions may or may not work in their context and the final question asked participants to identify a single solution to put forward. The desire for actionability also drove stakeholder selection; tenants on the CWG considered participant selection based on whether stakeholders were able to contribute ideas toward actionable solutions and had the power to make a contribution or commitment of some kind.

 After the DD, some tenants expressed that seeing change and action distinguished this project from previous projects that contributed to their mistrust. One expressed feeling heard because changes were made based on those discussions:

“*The message got through to the providers and the partners and the community more so than any of the dozens and dozens of meetings we’ve had over the years…Like, not just the vibe; like a pat on the back and some kind words to us little tenants for pacifying our engagement or needs or something, but just actually stuff being done” *(Tenant FG1).

 However, while tenants expressed the desire for “quick” and visible solutions, they also spoke poorly about “band-aid” solutions that do not address the underlying issue and solutions that demonstrate little effort and consistency, calling for solutions to which city housing and service providers could ensure commitment.

####  Tenant Narratives as Input

 Tenants typically used narratives as an expression of tacit knowledge when they contributed to the discussion or commented on decisions. Throughout CWG meetings tenants contributed numerous, detailed personal stories of their own first-hand experiences or those of other tenants. In multiple instances, these contributions impacted the direction of the conversation or led to a key decision, including topic selection and language use, eg, replacing “resident” with “tenant” in the issue brief because tenants did not feel a sense of community connectedness. Despite the frequency of tenant input in meetings, they rarely expressed direct disagreement or introduced new ideas without the framing of a narrative. In the few instances where tenants expressed forthright disagreement with another member of the CWG, it was directed toward another tenant.

###  Tenant Impact on Deliberative Discussions

Overall, the inclusion of tenants was described as crucial and important by every stakeholder group and “refreshing and enlightening” by a member of city housing. However, multiple factors impacting the course of discussions, both positively and negatively, were also identified. Table 6 displays an overview of results.

**Table 6:** Summary of Results of Tenant Impact on Deliberative Discussions

**1. Relationship Between Stakeholders**	a. Professional stakeholders gained new perspectives	*“I think as well it [the tenants’ participation] gave me insight more into tenant perceptions that I hadn’t had” *(Professional Stakeholder FG2). *“[The tenant voice] was also imperative to really understand and contextualise some of the issues, beyond just the day-to-day operations, but the actual more qualitative experiences of the residents” *(Professional Stakeholder FG2). *“I left there thinking like that… like, who else has to do all this work where they live just to feel comfortable where they live? Like, I felt like the tenants had this… Some of the tenants had this job, where they…there were expectations that they sort of have to fix this for everybody, and how do we help them?” *(Professional Stakeholder FG1).
	b. Power dynamics	Fear of retaliation *“[A]t the meetings you saw [a senior manager of city housing]… Various members of management were there. And when I’m speaking out and saying separate the housing from the social aspect […] and get an outside organization that’s qualified […] to worry about the buildings. […] they didn’t like that. And it kind of shows by them trying to, like, give me the heave-ho now. I’m gonna ’ fight it, and I’ll probably win, but… I should have a bag over my head and… and been more anonymous …[ the deliberative dialogue] has brought me to the forefront and to the attention of management. Like, the [senior manager of city housing] was kind of goin,’ right? High on his head and stuff” *(Tenant FG1). *“When you have tenants that are really struggling with something that’s going on in the building, and we can’t… we can’t get through to staff how sensitive this is, how incredibly sensitive this is, and you know, we’re not… we’re putting ourselves even on the line by discussing it here, in some respects, ‘ cause we just don’t know who’s who and who’s connected’” *(Tenant FG2). Hesitation to contribute/feeling of inadequacy *“I found [the mix of participants] unnerving, myself” *(Tenant FG1). *“I didn’t know how to speak to them ‘cause I’m not on their level, so I kinda ’ just withdrew a bit and listened more, which is what I’m doing now because I have a hard time understanding complex things” *(Tenant FG1).
**2. Dominance of Discussion**	a. Making space for tenant expression	Emphasis of negative experiences seen as less credible *“[L] ived experience shared so 'over the top' unusual difficult to feel this is seen as credible/believable and barrier to moving to action + much needed change” *(Tenant Survey Response). Some tenants dominated discussion *“I kinda ’ dominated the table, I feel, because I did have issues that I felt were never gonna ’ be heard” *(Tenant FG1). *“Some of them [the tenants] were… could have… you know, were a little overpowering. However, I think that that was, you know, the day… the day to have their voice, to be heard” *(Professional Stakeholder FG1). Service providers holding back to make space “*I didn’t contribute a whole amount through the deliberative dialogue just because I was in that experience of understanding the tenants needs, and I really wanted to be sure that I took that on board and… I mean, it affected me outside of that, but I don’t think that outside of providing minimal feedback, that I was giving too much back in terms of that, but I took so much away” *(Professional Stakeholder FG2).
	b. Emphasis of negative experiences	Perceived overrepresentation of negative experience *You know, being here on the ground, there are quite a number of tenants who actually enjoy being here [in this housing complex]. However, what seems to happen is that the ones who are showing up at these kind of events are the ones who are not happy with being here and are quite frustrated […] It seems that they’re probably not going to be happy with anything that is done around here. So, I don’t know how we would have got a more broader representation of the clients” *(Professional Stakeholder FG1). Tenant contributions brought conversation away from agenda *“I’m not sure that the gentleman at our table understood that we were to be discussing the possible solutions that were offered [in the issue brief]… the participant was still very active in the discussion, but he seemed to be getting… he was very off-topic. It had nothing…It was really not in relation to anything that we were discussing or the solutions that we were offering” *(Professional Stakeholder FG1).

####  New Perspectives

Professional stakeholders appreciated the new perspective of tenant experiences that was gained at the DD and the tenants felt encouraged seeing these stakeholders engaged and included in a project about listening to tenant voices. The DD offered a chance for service providers to receive constructive feedback from the tenants they serve and understand the burden and expectations placed on tenants in the building (see Table 6, 1a).

 It was evident that professional stakeholders understood the majority of the lessons learned and solutions reached through the experiences of tenants. In FGs, numerous service providers shared lessons learned at the DD by reiterating tenant narratives, rather than referring to the solution discussed or directly answering the question asked.

####  Power Dynamics

Power dynamics played a large role throughout the planning process and discussions in relation to feelings of tenant security. To mitigate these risks, researchers ensured that stakeholders such as general practitioners were not seated at small group tables with their own patients. However, building managers and social workers that serve the general population of the housing complex could not be separated from the tenants with whom they have direct contact or working relationships. Many tenants indicated that they freely spoke their minds regardless of who was at their table, but three tenants agreed that choosing to participate in the DD and FGs contributed to the risk of eviction and mistreatment in the building, based on their experiences with being outspoken in the past (Table 6, 1b).

Two tenants also felt unable to contribute meaningfully with professional stakeholders at the DD (see Table 6, 1b). In contrast, survey feedback indicated that many tenants attributed feeling prepared to discuss the issues meaningfully to the pre-DD orientation and one-on-one preparation with a member of the research team.

####  Making space for Tenant Expression 

Many participants, including some tenants, reported feeling that tenants dominated the small group discussions (Table 6, 2a). However, most stakeholders who reported this acknowledged that tenants had reasons for doing so and noted the value of tenants using their tacit knowledge and experience to lead discussions (Table 6, 2a). Some professional stakeholders reported that tenant contributions at times strayed from the agenda and issue brief, but acknowledged that this was appropriate due to their position and considered it a platform to air their frustrations.

 Two service providers and one public health stakeholder reported holding back their own insights during discussions to leave space and time for the input of tenants, noting that it was their “time to listen.” This, however, lead some public health and service provider stakeholders to feel they had less to contribute, as reflected in the FG and survey results.

####  Emphasis of Negative Experiences

It was a concern throughout the recruitment process that the isolated tenants whose input was most crucial would be the most difficult to recruit. This concern arose in DD discussions and in FGs, with tenants and professional stakeholders attributing some of the emphasis on negative experiences to a misrepresentation of broader tenant experiences (Table 6, 2b). One tenant shared that this emphasis of negative experiences made their positive experiences feel less heard and look less credible overall; multiple stakeholders expressed concern that airing frustrations brought the agenda off-track and away from solution-oriented discussions.

###  Tenant Impact on the Deliberative Dialogue Process

####  Conflict, Real and Perceived

 At times, conflicts arose between the goals or needs of the tenants and those of the research or researchers. One such conflict presented while determining the scope of the issue brief. The CWG discussed finding a balance between solutions that applied as closely (and therefore best) as possible to the current context and tenants, while remaining flexible enough to apply to rent-geared-to-income housing as a whole for future spread-and-scale. One tenant expressed concern that identifying and recording progress toward filling service gaps would inhibit the natural course of action, should a faster solution outside of the research be found.

 In some instances, researchers had to negotiate data collection approaches that were acceptable to the community. While it is ideal to record and collect as much relevant data as possible, particularly in a case study, it was important to find a balance between collecting valuable data and not making tenants feel too “studied.” Tenants reiterated negative past experiences with “selfish” research projects that appeared to only be concerned with fulfilling their own needs, highlighting the importance of demonstrating that this project was about more than just data collection and the tenants’ needs and desires would be accounted for throughout the process. A service provider brought to our attention early in the planning process that tenants in this housing complex felt extreme survey fatigue from numerous initiatives and so the post-DD survey was shortened to address only the most relevant key features.

####  Transparency

*Recruiting participants:*Some tenants in the FG admitted to being unclear about the DD’s purpose until attending the event. These tenants speculated that increasing awareness of the DD purpose in the general targeted community may have increased diverse participation by building awareness of and trust in the project.

*Reducing perceived conflict:* One tenant in the CWG perceived conflict between the needs of the tenants and goals of the researchers. While some conflicts, such as determining the appropriate amount of data collection, were necessary to navigate, researchers made repeated attempts to lessen the perception of additional conflicts. A presentation was given by researchers at the onset of the project, and frequently reiterated, about the goals, abilities, and roles of the research team in an attempt to mitigate these concerns.

*Managing outcome expectations:* It was reiterated throughout the planning process that key tenants did not trust the researchers because of three past research projects that “did not see it through.” Actionability arose as a major theme of the research project, and it was clear that tenants associated success of the partnership with visible action. However, what constitutes “action” and “change” may vary across stakeholders. During a FG, professional stakeholders explained that action was slow because partnerships needed to be formed and they wanted to ensure that the changes they committed to were stable, requiring small steps. Some managers explained that the output from the DD discussion alone was not enough to take the next steps:

“*Some of these [solutions] are, like, they’re not clear. So, it’s like, you know, ‘Pet Wall’ […] what does that mean? So, like, we’re trying…some things haven’t happened because we’re trying to still get from the tenants, like, ‘what does that mean?’ and we’re gonna start bringing one solution a month to those tenant planning drop-ins to be able to keep talking about where it’s at, what we’re doing, and then, what are we missing, what else does this look like and mean to you? But I think there’s some that we’re still trying to explore and kind of figure out what exactly tenants, and the people at the deliberative dialogue, meant by some of these” *(Professional Stakeholder FG3).

*Ethical exit: *The mistrust communicated by tenants on the CWG was rooted in concern that the research team would pull out before finishing the project or fulfilling its commitment to the community. Despite a review of the research team’s roles and disclosure that we would not be attending the CWG after the final DD debrief, one tenant felt that our departure left the community underserved, indicating that our intention to exit was not adequately communicated:

“*Now that this has been done […] [I’m] not sure if there will be follow-up for accountability; will someone come back to see how it went?... I saw it as being great from beginning, expanded more than I thought, and then abruptly stopped. Felt at the end we were just cut—end of today, that’s it. Like getting someone on a bicycle and letting him on his own, wobbly, and then not coming back to make sure he’s okay” *(Field notes, CWG).

## Discussion

Five main lessons and recommendations emerged from the results of this DD evaluation: *recognize diverse types of knowledge sharing*, *use facilitation to maintain balanced discussions, manage action-oriented outcomes, ensure transparency, *and *allow flexibility in the planning process*.

###  Recognize Diverse Types of Knowledge Sharing

Throughout the meetings, DD, and FGs tenants shared numerous personal stories and narratives. In comparison, professional stakeholders did not seem to share stories nearly as often or extensively. Such experiences have been documented by authors who involved the public or service users in research and ultimately provided recommendations to avoid meetings being overridden and agendas brought “off track” by expressions of personal experiences from the participants (eg, Brett et al^19^; Ong and Hooper^20^). However, while we did see evidence of tenants using the meetings, pre-DD orientation, DD, and FGs to air their frustrations, they acknowledged that it was because this might be their only chance to do so. Additionally, we speculate that these meetings and FGs were a safe space for tenants to discuss their challenges and feel heard. Given the hesitancy of tenants to trust that researchers and service providers were truly willing to listen and produce outcomes significant to them, it was especially important to allow tenants the space they needed to feel comfortable and express themselves openly, and this may be an integral step in establishing relationships and building relational quality.

 A number of key decisions made in the planning process could be traced back to personal stories shared by tenants about themselves or someone they knew and it appeared to be a method of expressing tacit knowledge. We therefore urge caution in discouraging or disregarding the significance of participants’ personal narratives, particularly because their relevance to and impact on the discussion was not always immediately clear. It is possible that tenants shared detailed stories to ensure that other stakeholders truly understood their living context. Professional stakeholders appreciated tenant inclusion for the express purpose of understanding situations from tenants’ perspectives. In FGs, stakeholders often repeated tenant stories as a means of expressing what they had learned, which may indicate that these narratives remain an integral part of understanding the concepts they represent, even when transferred to another stakeholder. To preserve these tools for public participants, researchers should be cautious about discouraging participation through personal narratives and must balance appreciation for this means of knowledge exchange while maintaining a strategy to stay close to the agenda. In addition, creating additional time in the pre-DD orientation and DD agendas for these narratives is recommended.

###  Use Facilitation to Maintain Balanced Discussions

 It was essential to maintain a balance between constructive input about barriers and solution-focused discussion, with the tendency of some tenants to dominate the discussion with personal experiences that strayed from the agenda. The engagement of a skilled facilitator is necessary to differentiate tangents from narrative contributions and recognize when feedback and experiences are not constructive to the issue at hand and return to the guidance of solutions in the issue brief.

Some stakeholders willingly held back their own insights to allow tenants more time to speak, as they also felt this DD was the tenants’ time to contribute and be heard. However, a DD is founded on the principle of diverse stakeholder input and transformative discussion.^2^ If one stakeholder dominates the discussion for any reason, the contextualization and tacit knowledge from other stakeholder groups are lost and vital barriers or facilitators to potential solutions may be overlooked. In turn, this impacts the next steps taken after the DD if not all barriers to implementation were sufficiently considered. It is therefore also important that facilitators maintain this balance by recognizing when stakeholders are voluntarily holding back for fear of overriding the discussion and encouraging their feedback throughout the conversation.

###  Manage Action-Oriented Outcomes

Unlike typical DDs, this dialogue was heavily focused on encouraging consensus in small groups and identifying actionable solutions that would lead to change. Typically, in DDs that do not aim for consensus, stakeholders rate the lack of consensus as a goal favourably and report that they appreciated the opportunity to consider and explore issues, recognizing that commitments could not be made on behalf of their organizations without further discussions.^3,7,8,21^ Professional stakeholders in this DD reiterated this sentiment, detailing the need to explore partnerships and better understand organizational capabilities before making commitments. However, actionability and tangible outcomes were vital in this context, as tenants had experienced numerous research projects that did not provide any positive change and insisted that outcomes from the research must be tangible and beneficial to them in order to maintain their trust and participation.

 This highlights the need for further discussions to take place after the DD. Service providers disclosed a need to reconnect with tenants to clarify the goal and intention of solutions as smaller steps were taken toward committing to the solutions overall. This aligns with tenants’ rejection of ‘quick fixes’ that are low-effort and non-committal. To sustain this action, it is necessary that there is oversight and structural collaboration to translate solutions into change.

Tenants equated being heard and the success of the project with tangible change and improvement, despite acknowledging that sustainable and beneficial solutions require planning and organizational commitment. Therefore, participants should be continually updated about the status of solutions including follow-up from researchers or professional stakeholders to demonstrate continued commitment. This follow-up communication with tenants may manage their expectations and allow them to see actionability *before,* and separate from, tangible change.

###  Ensure Transparency

 Understanding the context and history of the housing complex helped the researchers recognize where trust was most lacking and when transparency needed to be revisited throughout the project. The research team learned early in the planning process that the tenants had substantial mistrust in research projects and hesitancy to engage in or contribute to research. Although the research team exited within the planned timeframe and after fulfilling all planned and communicated commitments, one tenant viewed our exit as abandonment of the project. This demonstrated a need to be even more clear in our roles and abilities as researchers as well as the need to build a means of supported action and collaboration to hold professional stakeholders accountable to tenants while pursuing further action. The existing Steering Committee for the housing complex took this role on after the study was completed, with the service providers continually communicating progress on planned actions with the tenants.

###  Allow Flexibility in the Planning Process

All DDs vary in the design and implementation of key features, but researchers should be prepared to have extra flexibility when designing a DD with public participants. This is particularly important if they have experienced involvement fatigue from previous research projects or are a vulnerable population that is more likely to experience higher rates of chronic or mental illness that may impact the extent of project engagement.^22,23^ The research team must be prepared to address these needs and ensure that public participants have ample opportunity to define their own needs. For example, while most DDs hold two to three planning meetings (eg, Daya^6^; Boyko et al^5^), the CWG met five times and the Steering Committee three times to ensure that all necessary input from stakeholders was captured.

 A review of the community context demonstrated the importance of understanding pre-existing relationships and tensions within and between stakeholder groups that require ongoing navigation. With this contextual knowledge, we understood the importance of communicating our goals and abilities frequently as well as the importance of integrating actionability into the goal of the DD.

###  Strengths and Limitations

 This project has limitations that must be acknowledged. First, we recognize the “other” category in this analysis consists of a wide range of stakeholders. Thus, grouping these participants together might not be indicative of any substantial findings of trends for this group.

As described in some participant feedback, the sample of tenants who attended the DD may not have been representative of the tenant population as a whole due to sampling bias; people who tend to be more involved and social are more likely to accept invitations to participate.^24^ Because the DD focused on social isolation, the input of tenants who are the most socially isolated would have been valuable to determine what solutions are more likely to be accepted. Further, an overrepresentation of negative experiences in the building, as was speculated by some participants, could have led the discussion toward issues in the building that were not of concern to the majority of tenants.

 Finally, this project involved marginalized public participants with previous negative research experiences. While this research establishes the viability of public inclusion in DDs and a knowledge base to begin exploring the use of public participants in DDs more widely, the results from this study may not be reflective of nor transferable to all types of public participants who may be consulted in a DD. Future research should explore diverse community populations; in contexts where the issue and solutions are not as critical to the public participants’ lives, there may be a different emphasis placed on immediate actionability and a more balanced power dynamic among stakeholders.

This project also had numerous strengths. Case study methodology allowed descriptive data collection, attention to context, and heavily detailed reporting of the planning and decision-making processes, including factors that impacted the range of key DD features selected. We were able to look across the process to identify themes and impacts throughout all phases of the DD and draw direct correlations between the context and needs of the tenants, accommodations made in response, and the reception of those actions by all stakeholders. This descriptive account of the process is largely missing from the DD literature and may provide guidance to those looking for contextual similarities for transferability purposes. Additionally, we recorded and surveyed participants about many common DD features for comparability purposes with other studies.^8^

## Conclusion

 DDs can be used to bring together diverse perspectives, from professional and public participants, with the input of research evidence to tackle health and social issues. This case study emphasized the merits of including those with lived experiences in setting priorities and making decisions in their own community. However, to support a productive process, attention must be devoted to public participants’ needs and context before, during, and after the event. This study highlights the importance of collaborating with public participants during the planning phase of the DD to appropriately assess their needs and goals and anticipate necessary accommodations.

 Overall, the DD was rated positively by all participants and resulted in consensus of top-priority solutions for the community. All stakeholders responded positively to the inclusion of community tenants and deemed them essential to the process within the context of serving the targeted housing complex.

 Given the diversity between potential public groups that may be involved as stakeholders, future research is warranted on public participation in DDs throughout diverse contexts and communities.

## Acknowledgements

We are grateful for the support of the INSPIRE Core Working Group, the Steering Committee, Hamilton Public Health Services, and the participants of the deliberative dialogue. We gratefully acknowledge the contributions of Dr. Nancy Murray, the INSPIRE study research coordinator, who organized the planning, meetings, and data collection for the deliberative dialogue. This manuscript contains material pre-published in a master’s thesis, available at https://ir.lib.uwo.ca/etd/7362/.

## Ethical issues

 This research has been approved by Western University Non-Medical Research Ethics Board (113809) and the Hamilton Integrated Research Ethics Board (4631). Written informed consent was obtained by all members of the CWG and Steering Committee and all participants in the DD and FGs.

## Competing interests

 Authors declare that they have no competing interests.

## Authors’ contributions

 AK, RV, and RG contributed to the conception and design of this study and collected data along with TS. AK, RG, TS, and SLS contributed substantially to the analysis and interpretation of data and all authors contributed to drafting and critical revision of the manuscript.

## Supplementary files


Supplementary file 1. Description of Case Study Context.

Supplementary file 2. Deliberative Dialogue Agenda.

Supplementary file 3. Excerpt of Participant Survey.
