# Supplementary material for: Evaluating Public Participation in a Deliberative Dialogue: A Single Case Study
Source: Int J Health Policy Manag. 2022 Feb 28;11(11):2638–50. doi: 10.34172/ijhpm.2022.6588 (PMC9818103; doi:10.34172/ijhpm.2022.6588)
Supplement: Supplementary file 1 — Description of Case Study Context. [file ijhpm-11-2638-s001.pdf]

**Article title:** Evaluating Public Participation in a Deliberative Dialogue: A Single Case Study

**Journal name:** International Journal of Health Policy and Management (IJHPM)

**Authors' information**: Tiffany Scurr[1], Rebecca Ganann[2], Shannon L. Sibbald[1,3,4], Ruta Valaitis[2], Anita Kothari[1]*

(*Corresponding author: Email: akothari@uwo.ca)

[1]School of Health Studies, Faculty of Health Sciences, Western University, London, ON, Canada.

[2]School of Nursing, Faculty of Health Sciences, McMaster University, Hamilton, ON, Canada.

[3]Schulich Interfaculty Program in Public Health, Schulich School of Medicine and Dentistry, Western University, London, ON, Canada.

[4]Department of Family Medicine, Schulich School of Medicine and Dentistry, Western University, London, ON, Canada.

**Supplementary file 1.** Description of Case Study Context

## Table S1: Description of Case Study Context

The described context is based on tenant demographics captured in the Community Health Profile and descriptions of relationships throughout project documents.

| | |
|---|---|
| **Building demographics** | The rent-geared-to-income housing complex in which the INSPIRE project took place consisted of 565 tenants living in 555 units. The majority of tenants (80%) come from a precarious housing situation or homelessness and 9% were employed at the time of the study. Previously, the housing complex exclusively housed senior citizens, but the demographics of tenants has shifted – currently, 59% are aged 45 to 59 and 30% are aged 25 to 44. As the demographic of the housing complex shifts, tenants have more – and more advanced – needs, however the structure and regulations surrounding rent-geared-to-income housing has not changed with this shift, increasing the gaps between the needs of the community and the services provided. |
| **Physical environment of the housing complex** | Social isolation and tenant interactions with one another were largely shaped by the physical environment of the housing complex. Tenants reported issues with low quality repairs, mold, asbestos, bug infestations, and flood damage, which reduced their quality of life and increased the stigmatization toward the tenants of the building. Some tenants said that they felt ashamed and embarrassed to have friends and family visit them, and some have lost friends and family due to this lack of contact. Only 31% of tenants had regular contact with friends or family and 6% had diverse and frequent support.<br><br>Tenants also reported that violence, the presence of drugs, and bullies in the building kept many of them confined to their apartments. They reported constant disturbances during the night and graffiti in the hallways and elevators which led to feelings of an unsafe, unattractive, and insecure environment. In the CHP, only 23% reported feeling that the building was safe and secure overall. |

| | |
|---|---|
| **Mental health and addictions** | Although safety and security was being addressed by city housing, as they were in the process of hiring new guards and locking the exterior doors, the underlying issues have not yet been addressed for real progress to be made. These were identified as the mental illnesses and addictions that many tenants face – 1 in 2 and 1 in 3, respectively – that have been poorly addressed in favour of solving the symptoms of these problems.<br><br>Tenants felt that past solutions to addiction that city housing had implemented were more triggering than helpful because tenants were not consulted. For example, boxes for the safe collection of needles were installed in the building, but the presence of these boxes in unavoidable common spaces were triggering for some of those in recovery. |
| **Building culture** | Conflicts and tensions between groups of tenants contributed to a lack of socialization and success of initiatives that bring tenants together. Some past social programs that were not facilitated or supervised failed to foster socialization because large groups of tenants "clashed" with each other.<br><br>Some tenants felt that there were no consequences for tenants who commit violence and disturbances in the building, and as a result some tenants have taken it upon themselves to monitor the hallways and keep a record of disturbances. Other tenants respond negatively to these self-appointed roles, as they reported they did not like feeling watched by tenants who gave off a sense that they felt they were better than the others. Resentment was also directed toward tenants with officially appointed roles in committees, such as building attendants, because they felt that roles were given to more "popular" tenants who were not representative of the general tenant population. |
| **Mistrust of service providers and researchers** | In the last nine years, the building has been the subject of three research projects which the tenants reported as failed; either they lost funding and pulled out of the project before it was complete, or they gathered the information that they needed from the tenants to complete their research and exited the study without providing any results or findings that improved the tenants' lives. As a result, tenants felt used by past research projects and have lost hope that participating in research is worth their time and energy.<br><br>The tenants also lack trust in the service providers and building management. Tenants receive frequent surveys from building management and the purpose or results are rarely communicated back to them effectively, causing involvement fatigue and the impression that their efforts never truly inform decisions or lead to improvements. One tenant on the Core Working Group said that "there are a lot of promises about what improvements will be made but promises are never kept so people are rather skeptical." |
| **Access to resources in place** | Given these multiple drivers of inequality in the housing complex that burden the tenants more heavily than the population in the surrounding city, city housing has implemented resources in the building and nearby community, including on-site health services and social supports. However, less than half of tenants reported feeling comfortable accessing these resources and city housing reports that isolated tenants appear less likely to use these resources. |

**Legend: CHP = Community Health Profile**
