# Supplementary material for: Evaluating Public Participation in a Deliberative Dialogue: A Single Case Study
Source: Int J Health Policy Manag. 2022 Feb 28;11(11):2638–50. doi: 10.34172/ijhpm.2022.6588 (PMC9818103; doi:10.34172/ijhpm.2022.6588)
Supplement: Supplementary file 2 — Deliberative Dialogue Agenda. [file ijhpm-11-2638-s002.pdf]

Article title: Evaluating Public Participation in a Deliberative Dialogue: A Single Case Study

Journal name: International Journal of Health Policy and Management (IJHPM)

Authors' information: Tiffany Scurr[1], Rebecca Ganann[2], Shannon L. Sibbald[1,3,4], Ruta Valaitis[2], Anita Kothari[1]*

(*Corresponding author: Email: akothari@uwo.ca)

[1]School of Health Studies, Faculty of Health Sciences, Western University, London, ON, Canada.

[2]School of Nursing, Faculty of Health Sciences, McMaster University, Hamilton, ON, Canada.

[3]Schulich Interfaculty Program in Public Health, Schulich School of Medicine and Dentistry, Western University, London, ON, Canada.

[4]Department of Family Medicine, Schulich School of Medicine and Dentistry, Western University, London, ON, Canada.

Supplementary file 2. Deliberative Dialogue Agenda

AGENDA

INSPIRE Phase II Community Meeting (Deliberative Dialogue)	May 28th 2019<br><br>12:00pm – 4:30pm

**12:00pm – 12:30pm**	**Welcome and Lunch**	**30 minutes**
**12:30pm – 12:50pm**	**Introduction**<br>Opening Remarks and Agenda Overview<br>Rules of Engagement<br>Consent to Participate	**20 minutes**
**12:50pm – 1:30pm**	**Small Group – Session One**<br>Group One & Two: Communication<br>Group Three & Four: Engagement	**40 minutes**
**1:30pm – 1:35pm**	**Recess to Regroup**	**5 minutes**
**1:35pm – 1:55pm**	**Large Group – Session One**	**20 minutes**
**1:55pm – 2:10pm**	**Break**	**15 minutes**
**2:10pm – 2:50pm**	**Small Group – Session Two**<br>Group One & Two: Social Inclusion<br>Group Three & Four: Mental Health and Addiction	**40 minutes**
**2:50pm – 3:30pm**	**Large Group – Session Two**	**40 minutes**
**3:30pm – 3:45pm**	**Break**	**15 minutes**
**3:45pm – 4:00pm**	**Dotmocracy**	**15 minutes**
**4:00pm – 4:15pm**	**Survey**	**15 minutes**
**4:15pm – 4:30pm**	**Closing Remarks**<br>Outcome of the Dotmocracy<br>Overview of the Day	**15 minutes**
