# Supplementary material for: Evaluating Public Participation in a Deliberative Dialogue: A Single Case Study
Source: Int J Health Policy Manag. 2022 Feb 28;11(11):2638–50. doi: 10.34172/ijhpm.2022.6588 (PMC9818103; doi:10.34172/ijhpm.2022.6588)
Supplement: Supplementary file 3 — Excerpt of Participant Survey. [file ijhpm-11-2638-s003.pdf]

Article title: Evaluating Public Participation in a Deliberative Dialogue: A Single Case Study

Journal name: International Journal of Health Policy and Management (IJHPM)

Authors' information: Tiffany Scurr[1], Rebecca Ganann[2], Shannon L. Sibbald[1,3,4], Ruta Valaitis[2], Anita Kothari[1]*

(*Corresponding author: Email: akothari@uwo.ca)

[1]School of Health Studies, Faculty of Health Sciences, Western University, London, ON, Canada.

[2]School of Nursing, Faculty of Health Sciences, McMaster University, Hamilton, ON, Canada.

[3]Schulich Interfaculty Program in Public Health, Schulich School of Medicine and Dentistry, Western University, London, ON, Canada.

[4]Department of Family Medicine, Schulich School of Medicine and Dentistry, Western University, London, ON, Canada.

Supplementary file 3. Excerpt of Participant Survey

Table S3: Excerpt of Participant Survey

The survey questions that were asked of deliberative dialogue participants and that are relevant to this study are presented below. Additional questions appeared on the survey that were relevant only to the larger INSPIRE-PHC research study; those questions have been excluded with indication of their removal within the survey.

**Phase 2 *[Building Name]* INSPIRE Study Survey**			
1) Which category best represents your role?	[ ]	Community Resident	
	[ ]	Social services staff/manager/ director	
	[ ]	Public health staff/manager/ director	
	[ ]	Primary care staff/manager/ director	
	[ ]	Other. Explain:	________________

[Questions 2 through 7 removed for relevancy to this study]

8) The next statements are about the community meeting (deliberative dialogue) that took place about the *[building name]* project on May 28th 2019.		**Strongly Disagree**	**Disagree**	**Neutral**	**Agree**	**Strongly Agree**
8a	It was easy for me to get to (attend) the community meeting (deliberative dialogue).	[ ]	[ ]	[ ]	[ ]	[ ]
Please explain:		______________________________ ______________________________				
8b	I feel that I was well prepared to participate in the community meeting (deliberative dialogue).	[ ]	[ ]	[ ]	[ ]	[ ]

Please explain:		____________________<br>____________________				
8c	It was helpful to have the community meeting (deliberative dialogue) informed by the pre-circulated issue brief.	[ ]	[ ]	[ ]	[ ]	[ ]
Please explain:		____________________<br>____________________				
8d	The discussion at the community meeting (deliberative dialogue) was easy for me to understand.	[ ]	[ ]	[ ]	[ ]	[ ]
Please explain:		____________________<br>____________________				
8e	I feel that my ideas were heard at the community meeting (deliberative dialogue).	[ ]	[ ]	[ ]	[ ]	[ ]
Please explain:		____________________<br>____________________				
8f	I feel that my participation in the community meeting (deliberative Dialogue) was valued.	[ ]	[ ]	[ ]	[ ]	[ ]
Please explain:		____________________<br>____________________				
8g	I could easily read and understand all of the documents needed for me to participate in the community meeting (deliberative dialogue).	[ ]	[ ]	[ ]	[ ]	[ ]
Please explain:		____________________<br>____________________				
8h	In the community meeting (deliberative dialogue), the right people were involved to think about health and wellness for *[building name]* residents.	[ ]	[ ]	[ ]	[ ]	[ ]
Please explain:		____________________<br>____________________				
**[Questions 8i and 8j removed for relevancy to this study]**						
8k	It was helpful to have the opportunity to discuss different features of the problem, including (were possible) how it affects particular groups.	[ ]	[ ]	[ ]	[ ]	[ ]

Please explain:		____<br>____				
8l	It was helpful to have an engaged facilitator to assist with the community meeting (deliberative dialogue).	[ ]	[ ]	[ ]	[ ]	[ ]
Please explain:		____<br>____				
8m	It was helpful that the community meeting (deliberative dialogue) used a confidentiality code (participants know that the identity speaker will not be revealed) to allow for frank, off-the-record deliberations.	[ ]	[ ]	[ ]	[ ]	[ ]
Please explain:		____<br>____				
8n	It was helpful that consensus was encouraged in deliberative dialogue – community meeting.	[ ]	[ ]	[ ]	[ ]	[ ]
Please explain:		____<br>____				

9) On a scale of 1 to 10 circle what rating do you give the community meeting (deliberative dialogue) as a whole?	[1 – worst 10 - best]<br>1 2 3 4 5 6 7 8 9 10
10) What was good about the community meeting (deliberative dialogue)?	____<br>____
11) What could have been improved in the community meeting (deliberative dialogue)?	____<br>____

12) How did you feel about the following stakeholders' participation in the community meeting (deliberative dialogue)?

Policy Makers:

____

____

Service Providers:

____

____

Residents:

____

____

Researchers: ______________________________ ______________________________	
**[Question 13 removed for relevancy to this study]**	
14) Do you have any additional comments?	______________________________ ______________________________ ______________________________
Please explain:	______________________________ ______________________________ ______________________________
**Thank you for your time in completing this survey. Your input is valued.**	
